# A New Method of Supplying Artificial Teeth and Gums

**Published:** 1852-10

**Authors:** Wm. M. Hunter

**Affiliations:** Dentist


### * *A new Method of supplying artificial Teeth and Gums.*

BY WM. M. HUNTER, Dentist.

In the following pages I do not know that I shall give any thing new to a certain class of readers, but I feel convinced that the better informed of the practitioners in our profession, will find a practical elimination of good from old ideas.

To Delabarre must be given the credit of having first conceived and executed the union of artificial teeth already baked, with an artificial gum and plate, vide Fitch's Dental Surgery, 2d ed. Phil. 1835, which, I believe, contains the only English translation of that portion of his work.

To Audibran must we give the credit of having first made the claim, so far as I am informed, of having overcome the shrinkage of material, which claim was made in his published work, and was contested twenty years after by Lefoulon, but which principle is claimed by no other author. To Audibran I acknowledge my indebtedness for the idea of granulated body.

Desirabode and Lefoulon both give Delabarre credit for having done this kind of work, and publish his formula, the principle of which consisted in uniting a *flux* with the material used as an ordinary *base* or body, that it might fuse at a less heat than the teeth then in use.

Where is the new principle, in the patent claim now made? A flux is combined with what is technically termed a body or base, and the application is in every respect similar.

I stand upon the ground that I have perfected a body, (as applied to certain bodies and enamels made into artificial teeth by Jones, White and M'Curdy), which does not materially contract in the fire, and possesses more strength than any other body known to me, and which, with skilful handling, requires but one heat independent of the soldering of the teeth to the plate, to make perfect work.

It is applicable to the ordinary gold plate as used by dentists, generally in the form of *block-work*, and is made by me in continuous arches where a full denture is required, and it is equally applicable to cases where a few teeth are required, and can be fastened to the plate by soldering, riveting, or any other known method now in use. I also use it on an alloy of gold and platina, 20 and 22 carats fine, both in full arches and in partial sets. It is also applicable to platina plates for full arches, and as there has been a claim recently made for the application of platina for dental bases, I quote from Desirabode as published nearly thirty years ago. "It has not been a century since platina (vulgarly called white gold) has been known, and it has not been more than forty years employed by dentists. Its discovery and introduction in the arts has been a valuable resource because it possesses great consistence, although very malleable; and it is least of all mineral substances, affected by chemical agents and by buccal humors." The same author goes on to say that "M. Delabarre, finding that 20 carat gold and even platina alone are too ductile for certain purposes, and which he wished to have as solid as 18 carat gold, has proposed to make a double plate of platina which has all the solidity of the former." He still further goes on to describe the best methods of soldering platina, and speaks of the system pursued by other dentists of his day. So it will hardly do to set up the claim for novelty at this late date; the patentee however, is excusable, for as he does not read, he presumed that it was original in *the practice* from which he pilfered it, and so *originated* it.

Since I first put work into the mouth my modes have changed very much, my first efforts being made at a very high heat, thinking that a material of the requisite strength could not be made at a low (say the melting point of gold) heat to stand the fluids of the mouth, but which hypothesis I have found to be fallacious.

In 1849, I purchased receipts of Dr. E. Wildman of Philadelphia, whom I look upon as one of the most scientific men in the plastic department of our profession, which opened to me a new field of experiment and enabled me to perfect the work at a low heat; his *principles* of compounding and preparing being of great benefit to me.

I will now proceed to the description of the materials used, and the various compounds.

Silex should be of the finest and clearest description, and kept on hand ready ground, the finer the better.

Fused Spar should be the clearest Felspar, such as is used by tooth manufactures for enamels, completely fused in a porcelain furnace, and ground fine.

Calcined Borax is prepared by driving off the water of crystalization from the borax of commerce, by heating in a covered iron vessel over a slow fire, and it is better to use immediately after its preparation, as it attracts moisture. It should be perfectly clean and white, and free from lumps.

Caustic Potassa Optiumus. Known also as Potassa fusa.

Asbestos. Take the ordinary clean asbestos, free it from all fragments of talc or other foreign substances and grind fine, taking care to remove any hard fragments that may occur.

Granulated Body. Take any hard tooth material (I use the following formula: spar 3 oz., silex 1½ oz., kaolin ½ oz.) and fuse completely. Any very hard porcelain, wedgewood ware or fine china, will answer the same purpose. Break and grind so that it will pass through a wire seive No. 50, and again sift off the fine particles which will pass through No. 10 bolting cloth. It is then in grains about as fine as the finest gunpowder.

Flux. Upon this depends the whole of the future operations, and too much care cannot be taken in its preparation. It is composed of silex 8 oz., calcined borax 4 oz., caustic potassa 1 oz. Grind the potassa fine in a wedgewood mortar, gradually add the other materials until they are thoroughly incorporated. Line a hessian crucible (as white as can be got) with pure kaolin, fill with the mass, and lute on a cover a piece of fire clay slab, with the same. Expose to a clear strong fire in a furnace with coke fuel, for about half an hour, or until it is fused into a transparent glass, which should be clear and free from stain of any kind, more especially when it is to be used for gum enamels. Break this down and grind until fine enough to pass through a bolting cloth, when it will be ready for use.

Base. Take flux 1 oz. asbestes 2 oz., grind together very fine, completely intermixing. Add granulated body 1½ oz. and mix with a spatula to prevent grinding the granules of body any finer.

Gum Enamels. No. 1; flux 1 oz. fused spar 1 oz., English rose 40 grains. Grind the English rose extremely fine in a wedgewood mortar, and gradually add the flux and then the fused spar, grinding until the ingredients are thoroughly incorporated. Cut down a large hessian crucible so that it will slide into the muffle of a furnace, line with silex and kaolin each one part, put in the material and draw up the heat on it in a muffle to the point of *vitrifaction* not *fusion*, and withdraw from the muffle. The result will be a red cake of enamel which will easily leave the crucible, which after removing any adhering kaolin, is to be broken down and ground tolerably fine. It may now be tested and then (if of too strong a color) tempered by the addition of covering. This is the gum which flows at the lowest heat, and is never used when it is expected to solder.

No. 2. Flux 1 oz., fused spar 2 oz., English rose 60 grains. Treat the same as No. 1. This is a gum intermediate, and is used upon platina plates.

No. 3. Flux 1 oz., fused spar 3 oz., English rose 80 grains. Treat as the above. This gum is used in making pieces intended to be soldered on, either in full arches or in the sections known as *block-work*. It is not necessary to grind very fine in preparing the above formulas for application.

COVERING. What is termed covering, is the same as the formulas for gum, *minus* the English rose, and is made without any coloring whatever when it is used for tempering the above gums which are too highly colored, and which may be done by adding according to circumstances from 1 part of covering to 2 of gum, to 3 of covering to 1 of gum, thus procuring the desired shade. When it is to be used for covering the base prior to applying the gum, it may be colored with titanium, using from two to five grains to the ounce.

INVESTIENT. Take two measures of white quartz sand, mix with one measure of plaster of paris, mixing with just enough water to make the mass plastic, and apply quickly. The slab on which the piece is set should be saturated with water to keep the material from setting too soon, and that it may unite with it.

CEMENT. Wax 1 oz. rosin 2 oz. The proportions of this will vary according to the weather; it should be strong enough to hold the teeth firmly, and yet brittle enough to chip away freely when cold. A little experience will enable any one to prepare it properly.

Platina as usually applied I think objectionable, wanting stiffness; my method of using it is similar to that proposed by Delabarre, but possessing greater strength than even his method, and by it can be made as light as a good gold plate got up in the ordinary way. I first strike a very thin plate to the cast, and cut out a piece the size of the desired chamber, taking care not to extend it forward to embrace the palatal artery. Add wax to the plate for the depth of cavity, diminishing it neatly as it approaches the alveolar ridge. Cement this plate to the cast and take another metallic cast, strike another thin plate over the whole, and solder throughout with an alloy, of gold twenty-two parts, platina two parts, or with pure gold. The chamber thus formed is precisely the same as "Cleaveland's Patent Plate," but the space *between the plates*, for which he obtained his patent, is subsequently filled up, leaving a cavity resembling Gilbert's, but with a sharper edge when so desired. This space is filled up with base and enamel, and gives great stiffness without the ugly protrusion of the struck chamber. The plate thus formed assimilates much more closely to the palatal dome, not interfering with pronunciation; another great advantage gained by it is the impossibility of warping. I say *impossibility*, because I have submitted plates so constructed to the severest tests, and never had them to warp. It is well to rivet the two plates together before proceeding to solder, especially gold plates, and to bring the heat carefully upon them; once prepared there is no danger of change in the succeeding manipulations. I strike up the lower plate with a band on the labial edge about one sixteenth of an inch wide. This I do by trimming the wax impression before taking the plaster cast, or by building a ridge of wax on the plaster cast before taking the metal casts. Should the band (or turned edge) flare out too much, it may readily be bent in with a pair of pliers, etc. This style of work should not be applied except where the absorption may be said to be complete.

After the plates are perfectly adapted to the mouth, place wax upon each, which trim to the proper outline as regards length and contour of countenance, marking the proper occlusion of the jaws and the median line. These waxen outlines are called the *drafts* and are carefully removed from the mouth, and an articulator taken by which to arrange the teeth.

When the absorption is considerable and the plate in consequence is rather flat, it is necessary to solder a band or rim along the line where the upper draft meets the plate, about one sixteenth or one eighth of an inch wide, and fitting up against the outline of the draft. When the ridge is still prominent, the block will not of course be brought out against the lip so much, and a wire may be soldered on instead of the wider band. I think one or the other necessary, as it gives a thick edge to the block, rendering it far less liable to crack off than if it were reduced to a sharp angle; it also allows the edge of the plate to be bent in against the gum, or away from it, as circumstances may require, and afford in many cases a far better support for the plates than can be given to one in which the band is *struck up* or the edge turned over with pliers, where the block must extend to the edge of the plate. Some few cases do occur when the band may be struck as far back as the bicuspids with advantage, and some in the lower jaw where it is necessary to solder on the band, but the general practice is not so.

The upper teeth are first arranged on the plate antagonizing with the lower draft, supported by wax or cement, or both. Then remove the lower draft and arrange the lower teeth so that the coaptation of the cutting edges of the teeth shall be perfect as desired. The patient may now be called in again, and any change in the arrangement made to gratify his or her taste or whim. Now place the plates with the teeth thereon, on their respective casts, oil the cast below the plate and apply plaster of paris over the edge and face of the teeth and down on the the cast, say an inch below the edge of the plate. This will hold them firmly in their place while you remove the wax and cement from the inside, and fit and rivet backs to the teeth. When backed, cut the plaster through in two or more places, and remove. Clean the plate by heating. Cut the plaster so that while it will enable you to give each tooth its proper position, you can readily remove it from the teeth when they are cemented to the plate. Adjust the sections of plaster and the teeth in their proper positions. The plaster may be held by a piece of soft wire. *Cement* the teeth to the plate and strengthen the cement by laying slips of wood half an inch long along the joint and against the teeth. (I generally use the matches which are so plenty about the laboratory). Remove the sections of plaster, being careful not to displace any of the teeth. If it be intended to cover the strap with enamel, you should solder a wire after backing, and previous to replacing the teeth, along the plate parallel with the bottom of the straps, and about $\frac{1}{8}$ or $\frac{1}{4}$ of an inch from them.

The teeth are now backed and cemented to the plate and present an open space between the plate and the teeth, which is to be filled up with the base, using it quite wet to fill up the small interstices, filling in the rest as *hard and dry as possible.* Fill the cavity *between* the plates in the same manner, and oil the edge. Oil the surface of the base, envelope in the investient (precisely as you would put an ordinary job into plaster and sand for soldering) and set on a fire-clay slab previously saturated with water. When hard, chip away the cement, cooling it if necessary with ice, until it is perfectly clean. Along the joints place scraps and filings of platina very freely, and cover all the surface you wish to enamel with coarse filings, holding them to their place by borax ground fine with water. Apply pure gold as a solder quite freely, say two dwt. or more to a single set. Put in a muffle and bring up a gradual heat until the gold flows *freely*, which heat is all that will be needed for the base; withdraw and cool in a muffle. Remove the investient and fill up all crevices and interstices not already filed, with covering No. 2; cover the straps and base with the same, about as thick as a dime, and cover this with gum No. 2. about half that thickness. At the same time enamel the base in the chamber, and cover with thick soft paper. Set the plate down on the investient on a slab, with the edges of the teeth up. Fuse in the muffle and the work is completed. Blemishes may occur in the gum from a want of skill in the manipulation; should such occur, remedy by applying gum No. 1.

Should the patient object to the use of platina as a base, the work can be made as above on an alloy of gold and platina twenty carats fine, and soldered with pure gold, etc. as above. In all cases however, where it is used, the upper plate should be made as I have described above, but with platina any kind of plate can be used.

ORDINARY ALLOY. Blocks may be made and soldered to the ordinary plate if the absorption is sufficient to require much gum, without any platina. Arrange the teeth on wax on the plate, fill out the desired outline of gum and apply plaster $\frac{1}{4}$ of an inch thick over the face of the teeth, wax and cast. When hard, cut it into sections (cutting between the canines and bicuspids), remove the wax from the plate and teeth, bind the sections of the plaster mould thus made to their places with a wire, oil its surface and that of the plate, fill in the space beneath the teeth with the base, wet at first, but towards the last as hard and dry as possible, and thoroughly compacted. Trim to the desired outline on the inside, oil the base, and fill the whole palatal space with investient, supporting the block on its lingual side. Remove the plaster mould and cut through the block with a very thin blade between the canines and bicuspids. Take the whole job off of the plate, and set on a fire clay slab with investient, the edges of the teeth down; bring up the heat in a muffle to the melting point of pure gold. When cold, cover and gum with No. 3, gum and covering.

Another mode is to back the sections with a continuous strap (using only the lower pin), fill in the base from the front, use covering and gum No. 3, and finish at one heat. When the blocks are placed upon the plate, the other pin is used to fasten the gold back, which is soldered to it and the platina half-back; neither of these backs need be very heavy, as soldering the two together gives great strength and stiffness. Very delicate block work can be made in this way, and it is applicable also, where a few teeth only are needed.

A very pretty method, where a section of two or four teeth (incisors) is needed and only a thin flange of gum, is to fit gum teeth into the space, unite by the lower platina with a continuous back, and unite the joint with gum No. 3. A tooth left ungummed by the manufacturer would be best for the purpose. The same may be applied to blocks for a full arch, remembering not to depend entirely upon platina backs.

The method I prefer for full arches on ordinary plate, is to take a ribbon of platina a little wider than the intended base, and of the length of the arch, cut it nearly through in five places, viz: between the front incisors, between the lateral incisors and canines, and between the bicuspids. Adapt it to the form of the alveolar ridge with a hammer and pliers, and swage on the plate along where the teeth are to be set. Solder up the joints with pure gold, and proceed to back the teeth, &c. as before; making preparations for fastening, and removing the slip of platina from the gold plate before enveloping in the investient, when proceed as before.

When the teeth are arranged insert four platina tubes about one line in diameter, two between the molars and two between the cuspidati and bicuspids, and solder to the platina base. These are designed, after the teeth are finished, to be the means of fastening to the gold plate, either by riveting in the usual way, or by soldering pins to the gold plate passing up through the tubes, fastening with sulphur or wooden dowels. By these methods we are enabled to readily remove the block and repair it should it meet with any accident, and also in case absorption should go on, to restrike the plate, or to lengthen the teeth. The rim should be put on the gold plate after the block is finished, it gives great additional strength and a beautiful finish.

MEMORANDA. In preparing material always grind dry, and the most scrupulous cleanliness should attend all the manipulations. In all cases where heat is applied to an article in this system, it should be raised gradually from the bottom of the muffle and never run into a heat. Where it is desired to lengthen any of the teeth, either incisors or masticators, or to mend a broken tooth, it may be done with *covering*, properly colored with platina, cobalt or titantium.

In repairing a piece of work, wash it with great care, using a stiff brush and pulverized pumice stone. Bake over a slow fire to expel all moisture and wash again, when it will be ready for any new application of the enamel. Absorption occurring after a case has been some time worn, by allowing the jaws to close nearer, causes the lower jaw to come forward and drive the upper set out of the mouth. By putting the covering on the grinding surface of the back teeth in sufficient quantities to make up the desired length, the co-aptation of the denture will be restored, and with it the original usefulness.

Any alloy containing copper or silver should not be used for solder or plate, if it is intended to fuse a gum over the lingual side of the teeth, as it will surely stain the gum. Simple platina backs alone, do not possess the requisite stiffness, and should always be covered on platina with the enamel, and on gold with another gold back. In backing the teeth, lap the backs or neatly join them up as far as the lower pin in the tooth, and higher if admissible, and in soldering be sure to have the joint so made *perfectly soldered.*

As the work on platina plate presents fewer difficulties to the tyro, it would be well to gain experience upon that kind of work, before atempting its application to gold bases. The proper tooth for this work is not yet in the market, but I think will be ere long. A tooth finished at one heat by the manufacturer is best, although any tooth may be used that has been painted at a higher heat than the melting point of gold, being careful not to use any tooth in which gold may have been incorporated, as it will change color in the fire. A tooth with a natural shaped crown but thinner than the natural tooth, with the platina pins at a point that will allow of the back being covered without being clumsy, is wanted, and likewise a tooth resembling the natural tooth, except that the molars be made with one conical fang similar to a dens sapientiæ.

[We have been kindly furnished by Dr. Hunter in pamphlet form a general description of his method of supplying artificial teeth and gums. The directions are clear and explicit, and we doubt not the profession will hail with pleasure the new field which opens up for improvement in mechanical Dentistry. We award Dr. Hunter due praise in developing and bringing out his improvement, and also credit for his liberality in giving it in this form to the profession.

Some two or three pages of the close of Dr. Hunter's article, as in pamphlet form, we omit, as it has no reference to his method of supplying artificial teeth and gums; but has reference to a personal affair between himself and Dr. Allen, which we have so far kept out of the Register. This difficulty we leave where it should be; but would in all kindness remind the Dr. that his slur at the Mississippi Valley Association of Dental Surgeons, also at the "Ohio School" is unjust and needless.—Ed].

